# Introducing a single secondary alcohol dehydrogenase into butanol-tolerant *Clostridium acetobutylicum* Rh8 switches ABE fermentation to high level IBE fermentation

**DOI:** 10.1186/1754-6834-5-44

**Published:** 2012-06-28

**Authors:** Zongjie Dai, Hongjun Dong, Yan Zhu, Yanping Zhang, Yin Li, Yanhe Ma

**Affiliations:** 1 Department of Biochemistry and Molecular Biology, University of Science and Technology of China, Hefei, China; 2 Institute of Microbiology, Chinese Academy of Sciences, No.1 West Beichen Road, Chaoyang District, Beijing, 100101, China; 3 Graduate School of the Chinese Academy of Sciences, Beijing China

**Keywords:** *Clostridium acetobutylicum*, Butanol tolerance, Isopropanol-butanol-ethanol (IBE) fermentation, Secondary alcohol dehydrogenase, Acetone-butanol-ethanol (ABE) fermentation

## Abstract

**Background:**

Previously we have developed a butanol tolerant mutant of *Clostridium acetobutylicum* Rh8, from the wild type strain DSM 1731. Strain Rh8 can tolerate up to 19 g/L butanol, with solvent titer improved accordingly, thus exhibiting industrial application potential. To test if strain Rh8 can be used for production of high level mixed alcohols, a single secondary alcohol dehydrogenase from *Clostridium beijerinckii* NRRL B593 was overexpressed in strain Rh8 under the control of *thl* promoter.

**Results:**

The heterogenous gene *sADH* was functionally expressed in *C. acetobutylicum* Rh8. This simple, one-step engineering approach switched the traditional ABE (acetone-butanol-ethanol) fermentation to IBE (isopropanol-butanol-ethanol) fermentation. The total alcohol titer reached 23.88 g/l (7.6 g/l isopropanol, 15 g/l butanol, and 1.28 g/l ethanol) with a yield to glucose of 31.42%. The acid (butyrate and acetate) assimilation rate in isopropanol producing strain Rh8(psADH) was increased.

**Conclusions:**

The improved butanol tolerance and the enhanced solvent biosynthesis machinery in strain Rh8 is beneficial for production of high concentration of mixed alcohols. Strain Rh8 can thus be considered as a good host for further engineering of solvent/alcohol production.

## Background

*Clostridium acetobutylicum* is a Gram-positive, spore-forming anaerobe which is mainly used for acetone-butanol-ethanol (ABE) fermentation [1]. To improve the yield, titer, and productivity of ABE fermentation, various engineering strategies have been developed. These includes engineering central carbon flux redistribution [2-6], engineering regulatory mechanism [7,8], engineering phenotypic properties [9], and engineering butanol tolerance [10,11]. Of these strategies, engineering butanol tolerance has been demonstrated to be an effective approach to improve the productivity of ABE fermentation. For instance, introducing chaperon proteins *groESL* into *C. acetobutylicum* ATCC 824 [10] or introducing a glutathione biosynthetic pathway into *C. acetobutylicum* DSM 1731 [11] all resulted in an improved butanol tolerance and solvent productivity.

Improving butanol tolerance by random, non-rational engineering strategies can also lead to improved ABE productivity. *Clostridium* strain SA-1, a derivative of *Clostridium beijerinckii* ATCC 35702 obtained by serial enrichment can grow in the presence of 15 g butanol/liter and could produce 5-14% higher concentration of butanol in corn broth than that of the wild-type strain [12]. With the same method, *Clostridium* strain G1 showed a 40% increase butanol tolerance and a 17% improvement in the titer of butanol [13]. *C. acetobutylicum* EA 2018 has greater capability of solvent production, than the wild type strain *C. acetobutylicum* ATCC 824, especially for butanol, which was generated through butanol resistance screening of N-methyl-N-nitro-N-nitrosoguanidine treated *Clostridium* strain [14]. All these strains were obtained through native evolution or traditional mutagenesis. In our previous study, a butanol tolerant *C. acetobutylicum* mutant Rh8 was obtained by chemical mutagenesis and genome shuffling. This mutant showed a 46% improved butanol and 20% improved solvent titer, compared to that of the wild type strain DSM 1731 [15]. Characterization of strain Rh8 on the cytoplasmic and membrane proteome level revealed that strain Rh8 has developed a more stabilized membrane structure, a cost-efficient energy metabolism strategy, an earlier initiation of stress response mechanism, and strengthened solvent formation pathway [15,16]. These comprehensive characterizations suggest that strain Rh8 might be considered as an interesting host for further improvement of solvent productivity.

Recently, an alternative strategy to improve the cost-effectiveness for ABE fermentation was developed [17]. This strategy employs a secondary alcohol dehydrogenase [18] to convert acetone in ABE fermentation into a more expensive product isopropanol, thus switching ABE fermentation into IBE (isopropanol-butanol-ethanol) fermentation. Expression of a *sADH* gene from *C. beijerinckii* NRRL B593 into *C. acetobutylicum* ATCC 824 resulted in production of mix solvents (0.1 g/l acetone, 3.1 g/l isopropanol, 7.3 g/l butanol). The authors further constructed a finely engineered strain, where a synthetic acetone operon (*adc*, *ctfA*, *ctfB*) and *sADH* were overexpressed in a *buk* gene deletion mutant of *C. acetobutylicum* ATCC 824. The resulted engineered strain produced 20.4 g/l mixed alcohols (4.4 g/l isopropanol, 14.1 g/l butanol, and 1.9 g/l ethanol) in batch fermentation, with a yield to glucose of 0.3 g/g.

For a strain with industrial application potential, it is desirable to minimize the genetic manipulations. The well characterized butanol-tolerant mutant Rh8 strain showed a robust cellular structure and strengthened solvent biosynthesis, therefore can be considered as a good start point for engineering strains of industrial relevance. We investigated the potential of strain Rh8 using switch from ABE fermentation to IBE fermentation as a model. Surprisingly, we found simply introducing the *sADH* gene from *C. beijerinckii* NRRL B593 into strain Rh8 under the control of *thl* promoter resulted in a complete conversion of acetone into isopropanol, as well as a higher yield and higher titer of mixed alcohol in batch fermentations. The genetic operability and the capability of producing high level mix alcohols demonstrated that strain Rh8 is a potential good host for production of bio-based chemicals.

## Results

### Functional expression of secondary alcohol dehydrogenase in *C. acetobutylicum* Rh8

**Figure 1:**
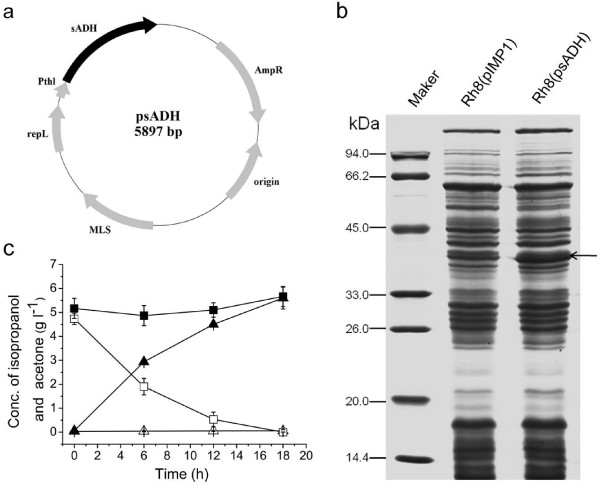
**Overexpression of *sADH *gene in *C. acetobutylicum *Rh8.** (**a**) Plasmid for expression of *sADH* gene in *C. acetobutylicum* Rh8. (**b**) SDS-PAGE analysis of overexpressing *sADH* gene in strain Rh8. The overexpressed protein sADH (theoretical molecular weight 37.65 kDa) is indicated by the arrow on the right. Strain Rh8(pIMP1) is the control strain containing empty vector. (**c**) Detection of sADH acitivty. *C. acetobutylicum* Rh8(psADH) and *C. acetobutylicum* Rh8(pIMP1) cells were cultured in RCM culture medium adding 5 g/l acetone when cells grow into exponential stage (OD_600_ = 0.8). The concentrations of acetone (solid squares/open squares) and isopropanol (open triangle /solid squares) in strain Rh8(pIMP1)/Rh8(psADH) were determined by HPLC.

To test the genetic manipulation feasibility of strain Rh8, both methylated and unmethylated shuttle vector pIMP1 were transformed into this mutant strain. The results showed that strain Rh8 could accept the methylated plasmid with normal transformation efficiency (10^4^ transformants per μg DNA). As to unmethylated plasmid, no transformant could be found on erythromycin plate. It means methlyating plasmid before transformation is still a necessary step for this strain. Previous work showed that the secondary alcohol dehydrogenase (encoded by *sADH*) from *C. beijerinckii* NRRL B593 could convert acetone into isopropanol in *C. acetobutylicum* ATCC 824 [17]. In that study, a late expression *adc* promoter was used, which might be the reason why there was little residual acetone still present in the culture medium. We cloned the secondary alcohol dehydrogenase gene (*sADH*) from *C. beijerinckii* NRRL B593 into pIMP1 under the control of the *thl* promoter. The methylated plasmid psADH was transformed into *C. acetobutylicum* Rh8. The overexpression of *sADH* gene was confirmed through SDS-PAGE analysis (Figure 1b). The cellular catalytic activity was detected by adding 5 g/l acetone into the RCM culture medium at exponential growth stage, then measuring the production of isopropanol with different time course. In contrast with the control strain, *C. acetobutylicum* Rh8(psADH) could completely convert the added acetone into isopropanol in 18 hours (Figure 1c). The results suggested that heterologous *sADH* gene was functional in *C. acetobutylicum* Rh8.

### Fermentation profiles of *C. acetobutylicum* Rh8(psADH) in CGM culture medium

**Figure 2:**
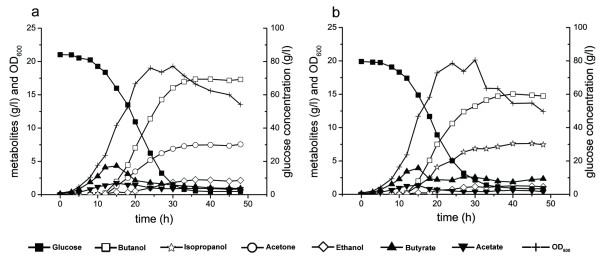
**Fermentation profiles of (a) *C. acetobutylicum *Rh8(pIMP1) and (b) *C. acetobutylicum *Rh8(psADH).** The fermentation was performed in CGM culture under pH 5.0 with 100 μg/ml erythromycin at 37°C in 7.5 L fermentor with an initial working volume of 3 L. Data shown represent the averages of two independent fermentations.

**Figure 3:**
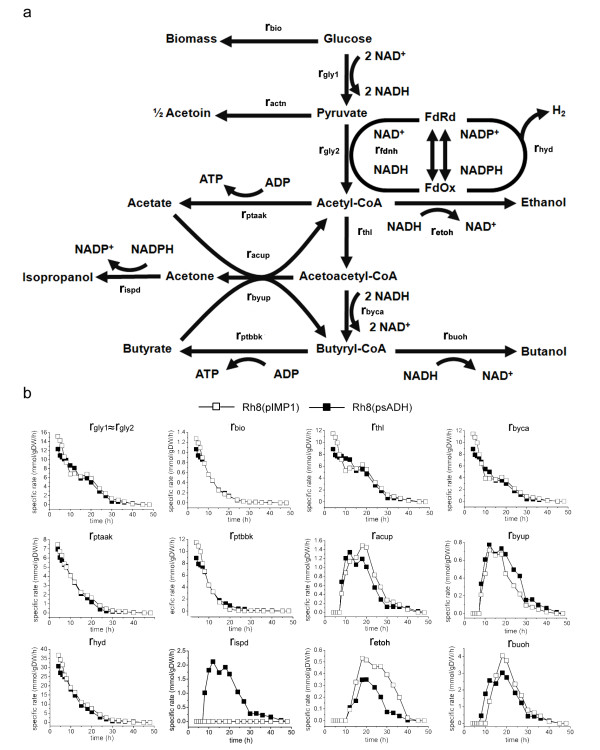
**GC-MS analysis the acetone in culture broth of *C. acetobutylium *Rh8(pIMP1) and *C. acetobutylicum *Rh8(psADH). **(**a**) The standard retention time of acetone was about at 2.0 min. (**b**) Detection of acetone in the culture broth of *C. acetobutylicum* Rh8(pIMP1). (**c**) Detection of residual acetone in the culture broth of *C. acetobutylicum* Rh8(psADH).

The *Clostridium* strain Rh8 has a stronger capability for ABE production compared with the wild type strain DSM 1731 [15,16]. The strain also showed a stronger IBE production capability, when introducing the secondary alcohol dehydrogenase (Table 1). Batch fermentations were performed in CGM culture medium under pH control (≥5.0) (Figure 2). The results showed that *C. acetobutylicum* Rh8(psADH) produced 7.6 ± 0.1 g/l isopropanol, 15 ± 0.1 g/l butanol and 1.28 ± 0.1 g/l ethanol (total solvent yield to glucose, 31.4%) after 48 h fermentation. Residual concentrations of acetate and butyrate were 0.5 ± 0.1 g/l and 2.2 ± 0.2 g/l, respectively. Notably, GC-MS data showed that there was almost no acetone (<0.02 g/l) in culture medium (Figure 3). The control strain Rh8(pIMP1) produced 7.5 ± 0.1 g/l acetone, 17.3 ± 0.2 g/l butanol, and 2.72 ± 0.1 g/l of ethanol (total solvent yield to glucose, 33.6%). The amount of residual acetate and butyrate were 0.82 ± 0.2 g/l and 0.93 ± 0.1 g/l. The maximum biomass of strain Rh8(psADH) was 6.03 ± 0.06 which was slightly higher than that of control strain (5.79 ± 0.06). The ratio of isopropanol and butanol to total solvent of strain Rh8(psADH) (0.95) was higher than that of control strain (0.9) (Table 1).

**Table 1:** **Comparison of major product profiles in pH-controlled (pH ≥ 5.0) batch fermentations of *C. acetobutylicum *** **strains**

**Characteristics and products**	**Strains**			
	**Rh8(pIMP1)**	**Rh8(psADH)**	**1731(pIMP1)**	**1731(psADH)**
Butanol	17.3 ± 0.2	15 ± 0.1	14 ± 0.2	12 ± 0.1
Isopropanol	ND	7.6 ± 0.1	ND	7.15 ± 0.1
Acetone	7.5 ± 0.1	ND	4.44 ± 0.1	ND
Ethanol	2.72 ± 0.1	1.28 ± 0.1	1.8 ± 0.1	1.5 ± 0.1
Butyrate	0.93 ± 0.1	2.2 ±0.2	1.8 ± 0.2	2.1 ± 0.2
Acetate	0.82 ± 0.2	0.5 ±0.1	3.5 ± 0.2	2.8 ± 0.2
Total solvent	27.52 ± 0.4	23.88 ± 0.3	20.24 ± 0.4	20.65 ± 0.3
IB or AB ratio^a^	0.9	0.95	0.91	0.93
Solvent yield	0.336	0.314	0.277	0.287

^a^(Isopropanol + butanol)/total solvent (g/g) or (acetone + butanol)/total solvent.

### Overexpression of the secondary alcohol dehydrogenase increases the acid assimilation rate and redistributes the reducing equivalent in *C. acetobutylicum* Rh8

**Figure 4:**
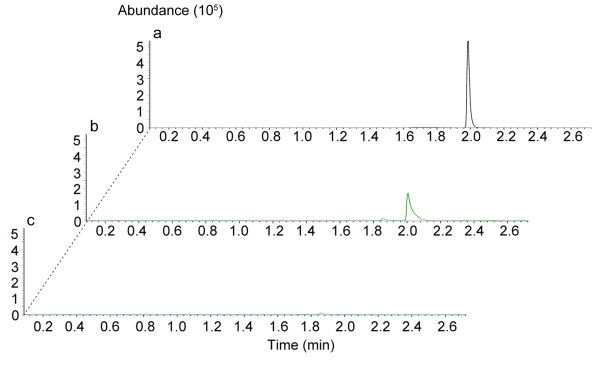
**Metabolic flux analysis of *C. acetobutylicum *Rh8(psADH) and *C. acetobutylicum *Rh8(pIMP1). **The detailed reaction formula used for flux analysis was shown in Appendix A.

The secondary alcohol dehydrogenase introduced into strain Rh8 is an NADPH dependent enzyme. To better understand the metabolic shift from ABE production to IBE production when overexpressing the secondary alcohol dehydrogenase, metabolic flux analysis (MFA) was performed to compare the flux profiles of central metabolic pathways between *C. acetobutylicum* Rh8(pIMP1) and *C. acetobutylicum* Rh8(psADH) (Figure 4).

MFA showed that the two strains have similar acid (acetate and butyrate) production rate in the first 20 hours of fermentation, and there was almost no acid production afterwards. However, the acetate assimilation rate of Rh8(psADH) was higher than that of the control strain from 8 to 15 h, and the butyrate assimilation rate of strain Rh8(psADH) was also higher. This suggested that the extended reaction from acetone to isopropanol increased the acid assimilation rate.

The growth rates (rbio) and glucose consumption rates (rgly1 and rgly2) of two strains were almost the same, indicating that expressing the *sADH* gene under the *thl* promoter did not introduce metabolic burden. The reactions of consuming reducing equivalent include rhyd, rthl, rbyca, rispd, retoh and rbuoh. Although the enzyme that we introduced is NADPH dependent, there were no significant differences on hydrogen production (rhyd) and on the flux from acetyl-CoA to butyryl-CoA (rthl, rbyca) between these two strains. However, the butanol and ethanol production strength (rbuoh, retoh) of strain Rh8(psADH) was lower than that of the control strain Rh8(pIMP1). This suggests that introducing the *sADH* gene leads to the redistribution of reducing equivalent among alcohols (isopropanol, butanol, and ethanol).

## Discussion

*C. acetobutylicum* Rh8 is a butanol-tolerant mutant with higher solvent production capability, which could convert 7–8 g/l acetone to isopropanol completely when overexpressing the secondary alcohol dehydrogenase under the control of *thl* promoter. Recently the *sADH* gene was integrated into *Clostridium acetobutylicum* chromosome under the control of *thl* promoter [19]. However, there was still 0.9 g/l residual acetone present in the medium, presumably due to the low activity of the secondary alcohol dehydrogenase, as only one copy of *sADH* was introduced. Another group expressed this gene with *adc* promoter in *Clostridium acetobutylicum* ATCC 824, using pIMP1 as a based vector. A small amount of acetone (<0.1 g/l) could be detected by GC in culture broth and the total residual concentration of acetone was amplified to about 1 g/l when *in situ* gas-stripping system was integrated with the fermentation [17]. The *adc* promoter was active during exponential growth, but maximal transcription from the *adc* promoter was reached in the exponential-growth later phase. Expression of the acetoacetate decarboxylase gene was turned off 2 h after reaching its maximum [20]. It means the expression of *sADH* gene would also turn off with the *adc* promoter. In this case, the residual enzyme would not be enough to convert the residual acetone to isopropanol. It is may be the reason why there was residual acetone in culture broth. We employed the most sensitive GC-MS to detect the presence of acetone in the culture broth of strain Rh8(psADH). The data showed that acetone was below the trace amount (<0.02 g/l) that the GC-MS could detect. This suggests that increasing the activity of the secondary alcohol dehydrogenase by strongly and perpetually expressing the *sADH* gene is a prerequisite to completely converting acetone to isopropanol.

Our previous work on the comparative analysis of cytoplasm proteome of strain DSM 1731 and Rh8 showed that ten proteins related to solvent formation, which are known to increase expression at the onset of solventogenesis, were differently expressed. Seven out of ten proteins (THL, AdhE1, CtfA/B, Adc, BdhA/B) in strain Rh8 that are involved in acetone and butanol production, significantly upregulated in acidogenesis, and the expression of these proteins were further increased in solventogenesis. While in the wild type strain DSM 1731, the expression of these seven proteins were only upregulated when cells entered solventogenesis. This might be the key why strain Rh8 produces higher level of solvents than that of the wild type DSM 1731. Notably, the expression level of acetone operon (*adc-ctfA-ctfB*) in strain Rh8, which is necessary for isopropanol production, was upregulated by a fold of 2.7 in acidogenesis compared with strain DSM 1731 [15]. We believe the significant upregulation of these solvent formation proteins contributed to strong capability of producing isopropanol and butanol by strain Rh8(psADH).

The production of butanol and ethanol by strain Rh8(psADH) showed a little decrease as compared to the control strain. This might be due to that the production of isopropanol competes with the production of butanol and ethanol for the reducing equivalent (NADH and NADPH). In *C. acetobutylicum* pyruvate-ferredoxin oxidoreductase could convert pyruvate into acetyl-CoA and carbon dioxide, producing reduced ferredoxin. This reduced ferredoxin could provide proton for production of NADPH, NADH, or hydrogen [21]. This is the major source for NADPH generation in *C. acetobutylicum*[22]. Nevertheless, no remarkable difference on the metabolic flux from glucose to acetyl-CoA (rgly1, rgly2) was found between strain Rh8(psADH) and strain Rh8(pIMP1). This suggests that the reducing equivalent produced by these two strains is in a similar amount. Since the secondary alcohol dehydrogenase introduced into strain Rh8 is an NADPH-dependent enzyme [18], isopropanol production will consume more reducing equivalent compared with strain Rh8(pIMP1). Therefore, it’s not surprising that the flux towards the NADH-dependent production of ethanol and butanol decreased, since the flux of another reducing equivalent consuming pathways (rthl, rbyca, rhyd) of these two strains remained similar (Figure 3). Another implication from this study is that the central pathway from glucose to butyryl-CoA is quite robust, since introducing an NADPH-dependent secondary alcohol dehydrogenase did not lead to notable disturbance on the consumption or generation of reducing equivalent of this central pathway. This understanding might be helpful when designing new engineering strategy for *C. acetobutylicum*.

## Conclusions

In this work, we introduce a secondary alcohol dehydrogenase into butanol-tolerant *Clostridium* strain Rh8. After 48 hours batch fermentation in glucose medium, this engineered strain could produce about 24 g/l mixed alcohol with yield of 31.4%. The potential of butanol-tolerant strain Rh8 was amplified by such simple metabolic engineering manipulation. Without downstream process, the total titer and yield of alcohol mixture was highest so far. It suggests that butanol tolerant strain is suitable host for further improving solvent or mixed alcohol production.

## Methods

### Bacterial strains, plasmids and culture conditions

All bacterial strains and plasmids used in this work are listed in Table 2. *C. acetobutylicum* Rh8, *C. acetobutylicum* DSM 1731 and *C. beijerinckii* NRRL B593 strains were stored in 15% (vol/vol) glycerol at −80°C. *E. coli* DH5α was used for cloning and vectors maintenance. *E. coli* ER2275 bears the methylating plasmid pAN1 for methylation plasmids [23]. *E. coli* strains were grown aerobically at 220 rpm and 37°C in liquid LB medium or on solidified LB medium supplementing with 1.5% agar. *C.* strains were grown in reinforced clostridial medium (RCM) [24] anaerobically at 37°C. Ampicillin and erythromycin were added at concentration of 100 μg/ml and 50 μg/ml if it is necessary. Cell density was determined by a UV-visible spectrophotometer (UV-2802PC; Unico, Shanghai, China) with the optical density at 600 nm. The dry cell weight (DCW) could be calculated from OD_600_ using an equation that DCW (g/l) = 0.3 × OD_600_[25].

**Table 2:** Strains and plasmids used in this study

**Strains and plasmids**	**Relevant charactristics**	**Soure or reference**
Strains		
*C. acetobutylicum* DSM 1731	Wild type strain	DSMZ
*C. acetobutylicum* Rh8	The mutant of *C. acetobutylicum* DSM 1731	[15]
*C. beijierinckii* NRRL B593	Contains *sADH* gene, wild type	NRRL
*C. acetobutylicum*	*sADH* expressing strain, harboring psADH	This study
Rh8(psADH)	Control strain harboring pIMP1	This study
*C. acetobutylicum*		
Rh8(pIMP1)		
*E. coli* JM109	*recA1 mcrB + hsdR17*	Lab storage
*E. coli* ER2275(pAN1)	Used for plasmid methylation before transformed into *C. acetobutylicum*	[26]
Plasmids		
pAN1	*ɸ3tI, p15a ori*, Cm^r^	[26]
pIMP1	The control plasmid, MLS^r^, Amp^r^, shuttle vector of *E. coli*-*C. acetobutylicum*	[26]
pITF	MLS^r^, Amp^r^, pIMP1 derivative for *fdh* expression under *thl* promoter	[27]
psADH	Used for *sADH* expression with *thl* promoter	This study

Abbreviations: Amp^r^, ampicillin resistance; Cm^r^, chloramphenicol resistance; MLS^r^, macrolide, lincosamide, and streptogramin B resistance; ɸ3tI, ɸ3TI methyltransferase gene of *Bacillus subtilis* phage ɸ3TI; thl, the promoter of thiolase gene in *C. acetobutylicum*; DSMZ, German Collection of Microorganisms and Cell Cultures, Braunschweig, Germany. NRRL, Agricultural Research Service Culture Collection, United State.

### DNA isolation, manipulation and transformation

The total genomic DNA of *C. beijerinckii* NRRL B593 and all the plasmids DNA in *E. coli* were isolated using an E.Z.N.A Plasmid Extraction Kit and E.Z.N.A Bacterial DNA Isolation Kit. PCR products and DNA fragments were purified with E.Z.N.A Cycle Pure Kit (Omega Biotek Inc., Guangzhou, China). All the enzymes were obtained from New England Biolabs (Beijing, China), and used according to the manufacturer’s protocols. *E. coli* ER2275 (pAN1) was used as host strain for methylating all plasmids before transforming them into *C. acetobutylicum* Rh8 and *C. acetobutylicum* DSM 1731. Methylated plasmids were electroporated into *C. acetobutylicum* by the method developed by Mermelstein [26].

### Construction of vector psADH

The gene encoding second alcohol dehydrogenase was cloned from the genomic DNA of *C. beijerinckii* NRRL B593, using primers sADH-1: 5’-CGCGGATCCATGAAAGGTTTTGCAATGCTAGGTATTTAATAAGTT-3’ and sADH-2: 5’-CCGGAATTCTTATAATATAACTACTGCTTTAATTAAGTC-3’. The resulted fragment was ligated into pITF [27] vector via *Bam*HI and *Eco*RI sites to construct psADH (Figure 1a) in which *sADH* gene was epxressed under control of consititutive *thl* promoter [27].

### Detection of sADH enzyme activity

When the cells were cultured into exponential stage of growth (OD_600_ = 0.8), acetone was added into the broth at a concentration of 5 g/l. The concentrations of acetone and isopropanol in the broth were assayed every 6 hours (0 h, 6 h, 12 h, 18 h) using High Performance Liquid Chromatography (HPLC).

### Cell free extracts preparation and protein expression analysis

The method used for protein expression assay in *C. acetobutylicum* was conducted as described previously [11] with slightly modification. The fresh cells were harvested by centrifugation and re-suspended in 1 ml TE buffer (pH 7.5). The cells were sonicated on ice for ten minutes using a Sonifier S-450D (Branson Ultrasonics Corp., Danbury, CT, USA) with the following protocol: 5 s sonication and 5 s intervals at 300 W. Cell debris was removed by centrifugation (12,000 g for 10 min at 4°C). The protein concentration in the rest cell free extract was qualified by using an RC DC protein assay kit (Bio-Rad) with bovine serum albumin as a standard. The same concentration protein was mixed with SDS-PAGE loading buffer and boiling for ten minutes. 10 μl samples were applied to a SDS-PAGE (12%) and visualized by Coomassie blue staining. The gel was scanned by Image Scanner 3 (GE Healthcare).

### Batch fermentation in CGM culture medium

Batch fermentations were performed in BioFlo 110 fermentors (New Brunswick Scientific, Edison, NJ) containing 3.0 L (working volume) of CGM [28] with 10% inoculum size, according to the fermentation method described in the literature [29], with slight modification. 8 ml refined corn oil was used as antifoam and 100 μg/ml erythromycin were added to the bioreactor together with 300 ml seed cultures (OD_600_ = 1.0). Experiments were duplicated and the presented data were the average values.

### High performance liquid chromatography (HPLC) analysis of metabolites

The main fermentation products (glucose, butanol, ethanol, acetone, isopropanol, butyrate, acetate) were determined using an Agilent 1200 high performance liquid chromatography (Agilent Technologies, Santa Clara, CA) with injection at volumes of 10 μl. An aminex HPX-87 H organic acid analysis column (7.8 × 300 mm) (Bio-Rad Laboratories, Inc, CA) was maintained at 15°C with 0.05 mM sulfuric acid as mobile phase and at a flow rate of 0.5 ml min^-1^. A refractive index (RI) detector (Agilent) was used for signal detection.

### Gas chromatography–mass spectrometry (GC-MS) detection for acetone

The residual acetone in culture broth was detected by GC-MS on a Agilent Technologies 6890 N GC-5973 N MSD. The GC was equipped with HP-5MS column (5%-phenyl-methylpolysiloxane as stationary phase; 30 m × 0.25 mm id × 25 μm film thickness, Agilent Technologies, Palo Alto, CA, USA). Helium (>99.999%) was used as carrier gas with a constant flow rate 1 ml/min. A 0.2 μl water samples was injected with split (100:1). The inject temperature was 150°C and the GC-MS transfer line temperature was 280°C, ion source 230°C, quadrupole 150°C. All compounds were analyzed with a nominal electron beam energy of 70 eV, and scan range was 15–75 amu. Following injection , the column temperature was held at 30°C for 5 min, then increased from 30°C to 150°C at 20°C/min. Compounds were identified by comparing their retention times with those of authentic reference compounds and the spectra with that of mass spectral libraries NIST02 (Rev. D. 04.00, Agilent Technologies, Palo Alto, CA, USA). Compounds were quantified by their selected ion abundances (acetone, ion m/z 43, m/z 58) relative to that of the standard.

### Metabolic flux analysis

The specific rates were calculated based on B-spline fitting according to previously work [30]. A stoichiometric model of *C. acetobutylicum* was derived from previously works with slightly modification [31]. It contains 16 metabolites and 15 reactions (See Appendix A). Combining with relations between acids uptake rates, a nonlinear objective (Eq. 1) was minimized to find an optimal rate distribution. The first item of the equation is a sum of weighted squared residuals and the second is the constraint on acids. Coding and calculation was implemented by using GAMS 23.6 with LGO solver.

$$\text{min}:f={||W^{-1}A\overset{\wedge }{r}-W^{-1}x||}^{2}+{\left(\text{rBYUP}{\left[\text{acetate}\right]}_{e}-0{\text{.315rACUP[butyrate}]}_{e}\right)}^{2}$$

## Appendix A

Pathway stoichiometry describing metabolism of solventogenic clostridia, as described previously [32]. Reversible reactions are indicated with ‘↔’ while irreversible reactions are indicated with ‘→’.IDFormula

$$r_{\text{bio}}{}^{\text{a}}:\text{ glucose }+0.873\text{ NADH }+\;14.85\text{ ATP}↔6\text{ biomass}$$

$$r_{\text{gly}1}:\text{ glucose}\to 2\text{ pyruvate }+\text{ 2 NADH }+\text{ 2 ATP}$$

$$r_{\text{gly}2}:\text{ pyruvate}\to \text{acetyl}-\text{CoA }+\text{ CO2 }+\text{ Reduced Ferredoxin}$$

$$r_{\text{actn}2}:\text{ pyruvate}\to \text{acetoin }+{\text{ 2 CO}}_{2}$$

$$r_{\text{etoh}}:\text{ acetyl}-\text{CoA }+\text{ 2 NADH}\to \text{ethanol}$$

$$r_{\text{ptaak}}:\text{ acetyl}-\text{CoA}↔\text{acetate }+\text{ ATP}$$

$$r_{\text{thl}2}:\text{ acetyl}-\text{CoA}↔\text{acetoacetyl}-\text{CoA}$$

$$r_{\text{acup}}:\text{ acetoacetyl}-\text{CoA }+\text{ acetate}\to \text{acetone }+{\text{ CO}}_{2}+\text{ acetyl}-\text{CoA}$$

$$r_{\text{byup}}:\text{ acetoacetyl}-\text{CoA }+\text{ butyrate}\to \text{acetone }+{\text{ CO}}_{2}+\text{ butyryl}-\text{CoA}$$

$$r_{\text{byca}}:\text{ acetoacetyl}-\text{CoA }+\text{ 2 NADH}↔\text{butyryl}-\text{CoA}$$

$$r_{\text{ptbbk}}:\text{ butyryl}-\text{CoA}↔\text{butyrate }+\text{ ATP}$$

$$r_{\text{buoh}}:\text{ butyryl}-\text{CoA }+\text{ 2 NADH}\to \text{butanol}$$

$$r_{\text{hyd}}:\text{ Reduced Ferredoxin}\to {\text{H}}_{2}$$

$$r_{\text{fdnh}}:\text{ Reduced Ferredoxin}↔\text{NADH}$$

$$r_{\text{ispd}}{}^{b}:\text{ acetone }+\text{ NADH}↔\text{isopropanol}$$

In biomass equation, NADH has been used as the single pool of reducing equivalents though reaction rispd is thought as NADPH-dependent. Essentially, all ATP produced is assumed to be consumed through growth and non-growth maintenance requirements. ATP stoichiometry was set to be 14.85 based on biomass composition equation in genome-scale metabolic model of *C. acetobutylicum* published before [33].^a^ The flux rbio was used as a measure of both growth and lysis by dynamically shifting the stoichiometric coefficients of glucose and NADH depending on the direction of rbio. For positive values of rbio, the indicated coefficients were used. For negative values of rbio, the coefficients of glucose and NADH were changed to 0.^b^ reaction catalyzed by *sADH* which is overexpressed in this work.

## Abbreviations

ABE: Acetone-butanol-ethanol; CGM: Clostridial growth medium; GC-MS: Gas chromatography–mass spectrometry; HPLC: High performance liquid chromatography; IBE: Isopropanol-butanol-ethanol; RCM: Reinforced clostridial medium; RI: Refractive index; sADH: Secondary alcohol dehydrogenase; SDS-PAGE: Sodium dodecyl sulfate polyacrylamide gel electrophoresis; TE: Tris-ethylenediaminetetraacetic acid.

## Competing interests

The authors have declared that no competing interests exist.

## Authors’ contributions

Conceived the experiments: ZD, HD, YZ, YL, YM. Performed the experiments: ZD, HD. Analyzed the data: ZD, YZ, HD. Contributed reagents/materials/analysis tools: ZD, HD, YZ. Wrote the paper: ZD, HD, YL. All authors read and approved the final manuscript.
